# A Cross-Sectional Study to Assess Capacity of Health Facility Laboratories in Zone One of Afar Regional State, Ethiopia

**DOI:** 10.1155/2018/9274127

**Published:** 2018-08-08

**Authors:** Chalachew Genet Akal, Tesfaye Andualem

**Affiliations:** Department of Medical Laboratory Science, Debre Tabor University, Debre Tabor, Ethiopia

## Abstract

Role of laboratory service in patient care is well recognized in developed compared to developing countries like Ethiopia where most medical decisions are based on clinical judgment. Laboratory based medical decisions save life and resources. Thus, health facilities (HFs) having capacitated laboratories provide better health care service. Thus, this study assessed capacity of laboratories found in all nine HFs in zone one of Afar region, Ethiopia. Cross-sectional study was conducted from January to May 2015. Data were collected using questioner from medical laboratory professionals (MLPs) and using cheek list from laboratory registration books. Collected data was analyzed using SPSS. Availability of laboratory related national guidelines and standard operating procedure varies among HFs studied. In 42 selected laboratory equipment and materials assessed, their availability shows variations among HFs from 13 (30.2%) to 32 (74.4%). Among different laboratory tests recommended to be performed at health center (HC) level by World Health Organization (WHO), six tests were not performed in all HC laboratories. Moreover, 11 (31.4%) MLPs received in-service training in the past one year. Internal quality control measures were done in 3 (33.3%) laboratories. However, all laboratories were involved in external quality assurance with varied schedules. Specimens after testing and specimen with prolonged transit time were discarded using standard rejection criteria in 8 (88.9%) and 3 (33.3) laboratories, respectively. Study showed that laboratories assessed have good capacities in involving external quality assurance and having proper laboratory test request format. But capacity of laboratories assessed is limited and unsatisfactory in number of laboratory tests performed at HC laboratories, in internal quality control measure implementation, and in getting up-to-date in-service training to staff. Thus, to enhance capacity of laboratories in HC, responsible bodies shall avail basic laboratory materials and equipment, make fair distribution of MLPs, provide up-to-date training, and implement internal quality control measures in laboratories.

## 1. Introduction

Laboratory services, either diagnostic or screening tests, are crucial parts of the health system having great contribution for infectious and noninfectious disease prevention and management [1]. The importance of accurate and reliable laboratory test results is well recognized in developed than developing countries [1–5]. Thus, majority of medical decisions are based on medical laboratory tests in developed countries. In USA, for example, laboratory test results influence around 70% of all medical decisions [5]. However, laboratory tests in developing countries are given little attention [6]; instead majority of treatment decisions are based on clinical judgment and empiric diagnoses [7]. Moreover, the International Health Regulations (IHR) adopted by World Health Organization (WHO) member states recommend to strengthen their notational capacity for surveillance as well as detection, assessment, notification, and response of disease outbreaks which in turn require capacitated laboratory services in health facilities [1].

In Africa, building the capacity of laboratory services in health facilities was given low priority for long time because of different challenges which include resource constrain, poor infrastructure, low qualified human resource, weak procurement and supply systems, lack of quality standards, limited quality assurance measures, poor laboratory management, and failure to implement/develop national laboratory policies [2, 4, 8, 9]. However, in the recent past, the need for building laboratory capacity is getting better attention [3, 10]. For this reason, there were efforts to enhance laboratory capacity over the past five years at national and regional level in many Sub-Saharan countries [11]. Moreover, a recent review by Olmsted et al. in 2010 suggests a general improvement in infrastructure and service provision in the region [12].

But as to our knowledge, the status of laboratory capacity in the study area was not studied. Studying the capacity of laboratories had a lot of significance which includes support proper laboratory resource allocation, indicates capacity gaps allowing different organizations working on the area to build laboratory capacities properly, and gives guidance for policy makers to strengthen the laboratory as well as health system at large. Therefore, objective of the present study was to assess the capacity of laboratories in health facilities found in zone one of Afar regional state, Ethiopia.

## 2. Methods and Materials

### 2.1. Study Design, Period, and Area

A cross-sectional study was conducted from January to May 2015 to assess the overall capacity of governmental health facility laboratories found in zone one of Afar regional state, Ethiopia. The Afar regional state is located in northeastern part of Ethiopia. The region, with total geographical area of 270,000 km^2^, shares common international boundaries with the State of Eritrea in the northeast and Djibouti in the east, as well as regional boundaries with Tigray Regional States in north-west, Amhara in the southwest, Oromia in the south and Ethiopian Somali in the southeast. The Afar National Regional State consists of 5 administrative zones (subregions) and 32 administrative units called Weredas [13].

### 2.2. Study and Sample Population

From 1.4 million total population in the region, nearly 87% of them are rural mainly dependent on pastoral and agropastoral livelihood systems. Zone one, with highest population in the region, has 10 governmental health facilities (one health post, 6 health centers, two hospitals, and one regional laboratory). Except the health post which did not have laboratory service, all nine health facilities were included in the study [13, 14]. Before data collection, ethical clearance as well as permission letter was obtained from Samara University health science college research ethical review committee and Afar regional health bureau, respectively.

### 2.3. Data Collection

Data were collected by 9 data collectors (diploma graduates in medical laboratory science) from medical laboratory professionals working in 9 health facility laboratories using structured questioner by adopting and modifying WHO laboratory capacity assessment tool [1]. Moreover, checklists were used to collect data from different laboratory registration books. Thus, using the questioner and checklists, different data were collected which include availability of common laboratory materials, equipment, reagents and laboratory stores as well as human resource profile, access to up-to-date in-service training, implementation of quality assurance measures, infection prevention measures, laboratory tests performed, and other related issues.

### 2.4. Quality Assurance and Analysis

To maintain the quality of data, pretest was done in one health center found in zone two of Afar regional state and the overall data collection was supervised by two supervisors. Moreover, every questioner was cheeked by supervisors for completeness just after data collection. The collected data were entered and analyzed using SPSS version 16.0 for different descriptive statistics.

## 3. Results

Based on assessments done on 42 selected laboratory equipment and materials, all nine health facility laboratories assessed had staining rack, drying rack, microscope slides, lancets, sharp container, and gloves in their laboratories. On the other hand, 66.7%, 55.6%, and 44.4% of health facilities did not have autoclave, differential counter, and hematocrit centrifuge in their laboratories, respectively [**Table 1**].

**Figure 1:**
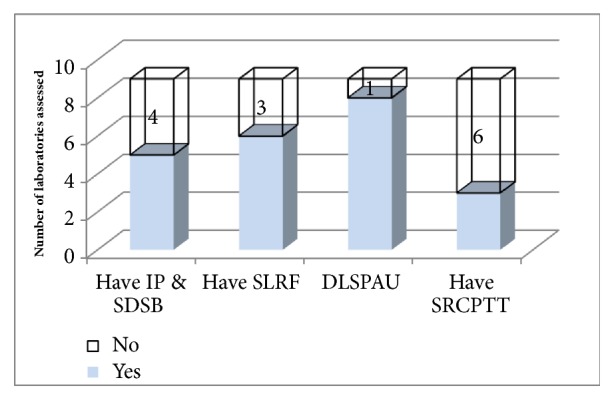
Guidelines, laboratory request form, specimen rejection, and disposal criteria availability in zone one of Afar region. Figure key: having IP and SDSB (having infection prevention and safe disposal of sharps and biohazard guideline), having SLRF (Standard Laboratory Request Form), DLSPAU (Discarding Laboratory Specimen Properly after Usage), and having SRCPTT (having specimen rejection, criteria, with prolonged transport time).

Though certain guidelines were available in assessed laboratories, none of them had guideline for destruction of damaged and/or expired products. Moreover, 66.7% of laboratories had standardized laboratory request format, whereas 3 (33.3%) laboratories assessed had criteria for discarding specimen with prolonged transport time [Figure 1]. All laboratories assessed report diseases to zone health office, regional health bureau, and Ethiopian ministry of health weekly, monthly, and quarterly based on the type of disease as per the country guideline.

In Ethiopia, there is an organized regular external quality assurance (EQA) program implemented in all health facility laboratories. Generally the hospital laboratories will conduct EQA programs on health center laboratories found in their catchment area. In turn the regional laboratories will conduct EQA on hospital laboratories in their catchment area. The national public health institute laboratory found in the country capital Addis Ababa will conduct EQA programs on the regional laboratories.

Majority of health facility laboratories (66.7%) assessed did not perform internal quality control measures like running controls daily, checking each reagent using known positive and negative controls, and counter check test reports with another colleague before dispatch. On the other hand, all health facility laboratories participated in the mandatory EQA program organized by the regional laboratory found in the region capital of Samara town (Ethiopia) and national public health institute of Ethiopia found in Addis Ababa. The EQA methods implemented by regional and national organizations include on site assessment and blind checking. After conducting both EQA methods, the organizations who conducted EQA will send feedbacks to the health facility laboratories based on the results. In the present study, the involvement of health facility laboratories in the mandatory national EQA program was regular in every three months. On the other hand, their involvement in the mandatory regional EQA program was not in regular base which varied from randomly performed to every 6-12-month period. The EQA measures were done in* Mycobacterium* spp. examination using acid fast staining and malaria diagnosis using Wright's or Giemsa stained blood film as well as in rapid serological tests of Human Immunodeficiency Virus (HIV) and to some extent to hepatitis B and C viruses. Moreover, 66.7% laboratories had different standard operating procedures.

**Figure 2:**
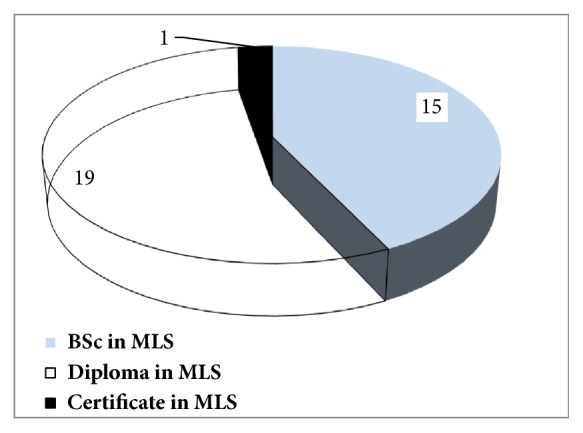
Staff profile working in nine health facility laboratories in zone one of Afar region, Ethiopia.** Figure key:** B.S. degree in MLS (B.S. degree in medical laboratory science), Diploma in MLS (Diploma in medical laboratory science), and Certificate in MLS (Certificate in medical laboratory science). Note: B.S. degree in MLS (which requires 4 years of university study in regular program), Diploma in MLS (which requires 2 years of university/college study in regular program), and Certificate in MLS (which requires 1 year of college study in regular program).

A total of 35 medical laboratory professionals were working in nine public health facility laboratories with different level of qualification [Figure 2]. Out of 15 medical laboratory technologists/scientists, 6 (40%) of them were in laboratories of two hospitals and in a regional laboratory. On the other hand, three health centers did not have any medical laboratory professional graduates with Bachelor of Science (B.S.) degree.

In the past one year, only 11 (31.4%) medical laboratory professionals received at least one training out of four in-service training sessions assessed in the study, namely, malaria, TB, HIV, and hepatitis. Six (54.5%) medical laboratory professionals attended training which were organized at national level where the remaining 5 (45.5%) training programs attended were organized at regional and zonal levels. the study also showed that in 5 (55.6%) health facility laboratories, the last laboratory supervisions done by either regional laboratory or national public health institute were before one month. But in the remaining laboratories, the last supervision was done in more than three months back.

Though all health facility laboratories had ministore or separate area within their laboratories for storing laboratory materials and reagents, record keeping of reagents delivered from store to laboratory for consumption was found only in 5 (55.6%) health facility laboratories. Moreover, there was no any system in 8 (88.9%) health facility laboratories for regular monitoring of material and reagent quantities available in their laboratory store. In 66.7% of health facility laboratories, there were a problem of obtaining adequate stock of laboratory reagents and equipment. The main reasons for under stock of materials and reagents were delay in ordering of laboratory reagents by health facilities form the central Pharmaceutical Fund Supply Agency (PFSA) in Addis Ababa and challenge in transportation system to deliver reagents from central to regional PFSA and health facility store in 55.6% health facilities studied followed by inconsistency of test demands by physicians (11.1%) and lack of information on how to obtain reagents and materials (11.1%).

Incineration using locally combustible materials (in 66.7%) and burial (in 33.3%) was used as methods for solid waste disposal. For liquid waste disposal, burial and others were used. In 66.7% of health facility laboratories, safety officer was present. There was no immunization offered for new staffs in any of health facility laboratories studied. In all laboratories studied, latex examination gloves were used as protective equipment.

Out of the nine health facilities studied, 2 were hospital and one was regional laboratory. The rest 6 health facilities were health centers. Based on WHO recommendation, 18 tests can be done at health center laboratory level. Among all six health centers studied, all of them do not perform 6 tests recommended to be performed at health center [Table 2].

## 4. Discussion

Different health care system stakeholders agreed that having laboratories with organized capacity to conduct different screening and diagnostic laboratory tests is one of the major inputs for providing quality health care system [3]. Published review and research articles conducted in assessing laboratory capacity are scant in Ethiopia except with specific study like malaria diagnostic capacity [15]. Along with few promising findings on the capacity of studied laboratories, the present study showed several areas of unsatisfactory results.

Only 22 (52.4%) materials and equipment assessed were found in all laboratories. Moreover, none of the laboratories had biosafety cabinet level-II as well as only three laboratories had basic laboratory equipment like Bunsen burner, autoclave, incubator, and ELISA. This finding agrees with a study done by Ishengoma DR et al. which reported that 43% of the laboratories did not have basic laboratory equipment [16]. Moreover, all laboratories in the present study failed to fulfill laboratory equipment recommended by WHO to be available at health center laboratory level. This might be explained that the study area had limited governmental and private health institutes and facilities which will assist in organizing the health facility laboratories.

Skilled medical laboratory professionals with the appropriate competencies and motivation are vital to the delivery of adequate laboratory services [2]. Among 35 laboratory professionals, 15 (42.9%) of them were laboratory technologist with B.S. degree where all of them were working in 6 (66.7%) health facility laboratories. Except unbalanced distribution, the present studies also indicated that health facility laboratories assessed had better qualified staff profile compared with a study done by Ishengoma DR et al. which reported that only 17% of staff were laboratory technologists [16]. The presence of better qualified staffs might be because of small health facility sample size included in the present study or it might be study time gap between the two studies. Moreover, this variation might indicate that the availability of qualified medical laboratory technologists is better in Ethiopia.

The present study also showed that only 31.4% of medical laboratory professionals received at least one out of four in-service training (on malaria, TB, HIV, and hepatitis topics) considered which is by far very low compared with the study by Hailegiorgis B et al. where 24% of laboratory professionals were involved in malaria microscopy training assessed [15]. This variation might be due to geographical (state) variation where there were limited health institutes giving training opportunity for medical laboratory professionals in the present study.

Laboratory quality assurance systems are critical to the success of any laboratory service [2]. With this regard, 33.3% laboratories assessed in the present study have internal quality assurance procedures which agree with a study done by Ishengoma DR et al. where 30% of the laboratories conducted internal quality control measures [16]. On the other hand, the study by Ishengoma DR et al. indicated that none of the laboratories had standard operating procedures (SOP) for quality assurance [16] unlike the present study where 66.7% of laboratories had SOP. This might be because of the participation of all laboratories in the present study in external quality assurance which may allow them to get SOPs from external assessors (the regional laboratory of Afar and national public health institute of Ethiopia). But this low internal quality assurance involvement of laboratories than external quality assurance in the present study might also indicate that there might be week follow-up, feedback, and supportive supervision given by responsible health personals from zonal and regional health office as well as from regional health bureau of the Afar regional state.

But the present study also showed that 66.7% of laboratories did not use standard criteria for discarding specimens with prolonged transit time. This finding, coupled with the very hot temperature of the study area, indicates that majority laboratories were not following the proper protocol to deliver laboratory service.

The infection prevention and control measures of laboratories were very low as it is being manifested in the availability of guideline on infection prevention, safe disposal of sharps, and safe disposal of biohazard medical waste only in 5 (55.6%) health facility laboratories. Moreover, all of the laboratories did not have guideline for destruction of damaged and/or expired laboratory materials and reagents.

## 5. Conclusion and Recommendation

Capacitated laboratories are crucial for accurate diagnosis [2]. The present study showed that the capacity of laboratories assessed was limited and unsatisfactory in the number of tests performed at health center laboratories, in internal quality control measure implementation, in the distribution of qualified medical laboratory professionals among different health facility laboratories, in getting up-to-date in-service training to staffs, in having the proper stock of laboratory materials and reagents in their stores, and in having basic laboratory equipment and material. On the other hand, the study also showed that health facility laboratories assessed have a promising capacity in their involvement in external quality assurance, in availability of different guidelines (though not uniform), in total number of qualified medical laboratory professionals, in the availability of laboratory request forms, and in availability of separate stores in health facility laboratories. Finally we recommend the zonal health office, the regional health bureau, the regional laboratory of Afar regional state as well as Ethiopian public health institute and Ethiopian ministry of health to build the capacity of laboratories found in zone one of Afar regional state in terms of availing basic laboratory materials and equipment so as to make number of tests performed to the WHO recommended level, make fair distribution of laboratory professionals, provide up-to-date in-service training, give supportive supervision on internal quality control measures, and maintain proper stock of laboratory reagents and materials.

## Figures and Tables

**Table 1:** List of laboratory equipment and materials found in zone one governmental health facility laboratories of Afar region, Ethiopia.

**No **	**Equipment list**	**Number of laboratories having equipment (**%**)**
1.	Autoclave	3 (33.3%)
2.	Microtome disposable blade	5 (55.6%)
3.	Automatic micro pipettes	4(44.4%)
4.	Pasteur pipettes	7 (77.4%)
5.	Bunsen burner (with gas cylinder)	3 (33.3%)
6.	Spirit lamp	6 (66.7%)
7.	Chemistry auto analyzer	2 (22.2%)
8.	Deep freezer (-20°C)	2 (22.2%)
9.	Desktop computer & printer (office)	4(44.4%)
10.	Differential counter	4(44.4%)
11.	ELISA reader and washer	2 (22.2%)
12.	Flow cytometer CD4 or viral load instrument	2 (22.2%)
13.	Hematology auto-analyzer	4(44.4%)
14.	Incubator	2 (22.2%)
15.	Binocular light microscope	9 (100%)
16.	Laboratory refrigerator (electric operated)	9(100%)
17.	Hemaglobinometer	7 (77.8%)
18.	Bench top electric centrifuge	8 (88.9%)
19.	Haematocrit centrifuge	5 (55.6%)
20.	Manual centrifuge	0 (0%)
21.	Blood mixer	4(44.4%)
22.	Class II biosafety hood	0 (0%)
23.	Haemocytometer	7 (77.8%)
24.	VDRL shaker	4 (44.4%)
25.	Water bath	2 (22.2%)
26.	Thermometer (-20°C)	9 (100%)
27.	Slide staining rack	9 (100%)
28.	Slide drying rack	9 (100%)
29.	Microscope slides	9 (100%)
30.	Slide troughs	9 (100%)
31.	Slide box	9 (100%)
32.	Tally counter	4 (44.4%)
33.	Beakers	5 (55.6%)
34.	Flasks	5 (55.6%)
35.	Funnels	5 (55.6%)
36.	Wire loop with holder	6 (66.7%)
37.	Lancets	9 (100%)
38.	Sharps container	9 (100%)
39.	Biohazard container	6(66.7%)
40.	Gloves	9 (100%)
41.	Timer	8 (88.9%)
42.	First aid kit	6 (66.7%)

**Table 2:** Tests performed at six health centers laboratories of zone one of Afar region, Ethiopia.

No	Laboratory Test۩	Number of health centers laboratories (%)
1.	Hemoglobin estimation	2 (33.3 %)
2.	Blood slide for haemoparasites	6 (100%)
3.	Stool microscopy for parasites	6 (100%)
4.	Sputum for AFB	6 (1000%)
5.	Skin slit for AFB	0 (0%)
6.	Urine sediment microscopy	6 (100%)
7.	Urine chemical test using multiple urine deep stick	6 (100%)
8.	Syphilis screening	5 (83.3%)
9.	Sickle cell screen	0 (0%)
10.	Genito-urinary tract specimens	0 (0%)
11.	Pus swabs	0 (0%)
12.	HIV screening	6 (100%)
13.	Blood grouping (ABO)	6 (100%)
14.	Rhesus typing (Rh blood grouping)	6 (100%)
15.	Total white cell count	2 (22.2%)
16.	Differential white cell count	3 (33.3%)
17.	Cerebrospinal fluid microscopy	0 (0%)
18.	Cerebrospinal fluid chemistry	0 (0%)

## Data Availability

The data used to support the findings of this study are available from the corresponding author upon request.

## Abbreviations

BSc: Bachelor of Science
HIV: Human immunodeficiency virus
IHR: International Health Regulations
SOP: Standard operating procedure
WHO: World Health Organization.
